# Comparacão dos Parâmetros Ecocardiográficos Convencionais e com *Speckle Tracking* entre Indivíduos Saudáveis e Transplantados Cardíacos sem Rejeição

**DOI:** 10.36660/abc.20230681

**Published:** 2024-07-25

**Authors:** Aline Oliveira Martins Campo Dall’Orto, Maria Estefania Otto, Simone Ferreira Leite, Marco Antônio Freitas de Queiroz Maurício, Natália Taveira Martins, Samuel Rabelo Araújo, Soraya Vasconcelos Almeida, Luiza Valle Oliveira Brizida, Fernando Antibas Atik

**Affiliations:** 1 Universidade de Brasilia Brasília DF Brasil Universidade de Brasilia, Brasília, DF – Brasil; 2 ICTDF Brasília DF Brasil Instituto de Cardiologia e Transplantes do Distrito Federal (ICTDF) , Brasília, DF – Brasil

**Keywords:** Transplante de Coração, Ecocardiografia, Função Ventricular

## Abstract

**Fundamento:**

A ecocardiografia é essencial para avaliação do coração transplantado. No entanto, os valores de normalidade no transplante cardíaco (TC) não estão claramente definidos. Objetivos: Comparar parâmetros ecocardiográficos convencionais e pela técnica de *Speckle Tracking* entre pacientes transplantados cardíacos sem rejeição e uma população de indivíduos saudáveis.

**Métodos:**

Foram estudados prospectivamente pacientes adultos, com menos de 1 ano de TC, que realizaram biópsia endomiocárdica de vigilância seguido de ecocardiograma transtorácico (ETT). Medidas convencionais de ETT acrescidas da avaliação de mecânica cardíaca por meio do *Strain* pelo *Speckle Tracking* foram realizadas e comparadas com um grupo de voluntários saudáveis. A significância estatística adotada para o estudo foi de 5%.

**Resultados:**

Avaliou-se 36 pacientes transplantados sem rejeição, os quais foram comparados com 30 indivíduos saudáveis. Observou-se redução nos valores de *Strain* Global Longitudinal de Ventrículo Esquerdo em valor absoluto (11,99% transplantados, 20,60% controle, p<0,0001), *Strain* de parede livre de Ventrículo Direito (transplantados 16,67%, controle 25,50%, p<0,0001) e dos índices de trabalho miocárdico (p<0,0001), maior tamanho do átrio esquerdo (38,17 ml/m^2^ transplantados, controle 18,98 ml/m^2^, p<0,0001), maior índice de massa e espessura relativa das paredes (p<0,0001) e a presença da Doença de Chagas como principal etiologia para o transplante.

**Conclusão:**

Os transplantados cardíacos estáveis e sem rejeição apresentaram diferenças com relação aos parâmetros ecocardiográficos comparados com indivíduos saudáveis. Estes achados indicam que medidas ecocardiográficas convencionais e de mecânica cardíaca são alteradas em transplantados mesmo na ausência de rejeição e podem ser relevantes para o contexto clínico e acompanhamento dos pacientes.

## Introdução

A ecocardiografia é uma ferramenta útil e de fácil realização para avaliação do coração transplantado em várias etapas do transplante cardíaco (TC), desde o ato intra-operatório até avaliações seriadas no pós-operatório, além do controle de complicações após biopsias endomiocárdicas, acompanhamento na rejeição celular aguda e diagnóstico de isquemia por doença vascular do enxerto.^1^

A normalização da função ventricular esquerda após a realização do TC é responsável pela melhora dos sintomas e apresenta um grande impacto no prognóstico,^1^ sendo a fração de ejeção, avaliada por meio do ecocardiograma, método consagrado para estimativa da função ventricular.^2^

Mais recentemente, descreveu-se uma nova modalidade de ecocardiograma bidimensional denominada *Speckle Tracking* , a qual tem a habilidade de detectar disfunção ventricular esquerda antes de alteração na fração de ejeção, por meio da análise da deformação miocárdica ( *strain* ).

Adicionando a medida da pressão arterial a este método, é possível determinar o trabalho miocárdico. Esta nova variável, mostrou superioridade ao *Strain* Global Longitudinal na avaliação da demanda de oxigênio e função cardíaca, já que considera a deformação miocárdica conjuntamente com a pós-carga.^3^

Em indivíduos normais, mudanças na função ventricular cardíaca estão bem descritas por meio de valores de normalidade compilados em diversos estudos,^2 , 4^ contudo para os pacientes transplantados, ainda é escassa a informação sobre o desempenho normal do enxerto por evidências científicas limitadas nas pesquisas disponíveis.^5^

Deste modo, este trabalho tem como objetivo comparar parâmetros ecocardiográficos entre pacientes transplantados cardíacos sem rejeição e uma população de indivíduos saudáveis e, dessa forma, observar o valor médio do *Strain* Global Longitudinal do Ventrículo Esquerdo (SGL VE) pelo *Speckle Tracking* e de parede livre do Ventrículo Direito ( *SL* VD), além dos índices de Trabalho Miocárdico nas duas populações.

## Métodos

### População

O cálculo do tamanho da amostra foi baseado em estudos que compararam SGL VE em pacientes transplantados.^6 , 7^ Dessa forma, adotou-se um poder de 80% para detectar uma redução de 10% do valor do SGL VE, considerando o normal 21% (valor absoluto) e o desvio padrão de 4%, com um erro alfa de 5%. Em estudo anterior publicado pelos autores^7^está descrita a análise completa do cálculo da amostra. Este estudo comparou o grupo de pacientes com e sem rejeição. No entanto, para comparação de pacientes sem rejeição e voluntários saudáveis, o número necessário foi de 25 indivíduos em cada grupo e para comparação entre transplantados com e sem rejeição foi de 34 pacientes em cada grupo. Dessa forma, resultou em 36 pacientes sem rejeição na biópsia e 30 pacientes saudáveis (valor acima de 25).

De janeiro de 2017 a dezembro de 2019, foram estudados prospectivamente pacientes adultos maiores de 18 anos e com menos de 1 ano de TC em um hospital de referência.

Foram incluídos ao mesmo tempo um grupo de voluntários saudáveis e sem comorbidades que aceitaram participar do estudo. Estes foram submetidos a exames laboratoriais básicos, eletrocardiograma, bem como medida da pressão arterial não invasiva previamente ao ecocardiograma.

Os critérios de exclusão para os pacientes transplantados foram: fração de ejeção do ventrículo esquerdo (FEVE) abaixo de 53%, reativação por Doença de Chagas, rejeição celular aguda identificada por biópsia em algum momento do acompanhamento, rejeição humoral, ritmo cardíaco irregular, doença vascular do enxerto confirmada, janela acústica limitada e aqueles que se recusaram a participar do estudo.

Esta pesquisa foi aprovada pelo Comitê de Ética e sua inscrição na Plataforma Brasil é o número 65910517.0.0000.0026. Todos os participantes do estudo assinaram o termo de consentimento livre e esclarecido.

### Biópsia endomiocárdica

Os pacientes transplantados estavam em seguimento de rotina e foi realizado biópsia endomiocárdica (BEM) de vigilância seguida de ecocardiograma transtorácico (ETT) no mesmo dia, com intervalo entre os exames inferior a 4 horas. O protocolo de BEM de vigilância consiste em realização de biópsia uma vez na semana para o primeiro mês, a cada 15 dias no segundo mês e mensalmente de 3 a 7 meses após o TC, totalizando 9 biópsias em 6 meses. Após 7 meses, uma cintilografia miocárdica é realizada quando disponível e a biópsia é realizada apenas quando a cintilografia é positiva para inflamação.

A BEM foi realizada invasivamente por punção da veia femoral e realização de fluoroscopia, com 3 a 5 fragmentos coletados na região do septo interventricular, acessado pelo ventrículo direito. As amostras foram analisadas através de microscopia óptica após serem fixadas em hematoxilina e eosina.^8^ O patologista responsável pela análise da biópsia desconhecia os resultados ecocardiográficos e as alterações encontradas nos fragmentos foram classificadas de acordo com o sistema de graduação que avalia rejeição da Sociedade Internacional de Transplante Cardíaco e Pulmonar ( *International Society for Heart and Lung Transplantation* -ISHLT), de 2004.^9^

Os pacientes transplantados foram divididos em dois grupos de acordo com o resultado histopatológico da BEM: Grupo 1: Sem Rejeição Celular Aguda (graus 0 e 1R) e Grupo 2: Com Rejeição Celular Aguda (graus 2R e 3R). Os pacientes do grupo 2 foram excluídos da análise e os pacientes do Grupo 1, que são os transplantados cardíacos sem rejeição, foram comparados com indivíduos saudáveis que compunham o grupo controle ( Figura Central ).

**Figura Central:**
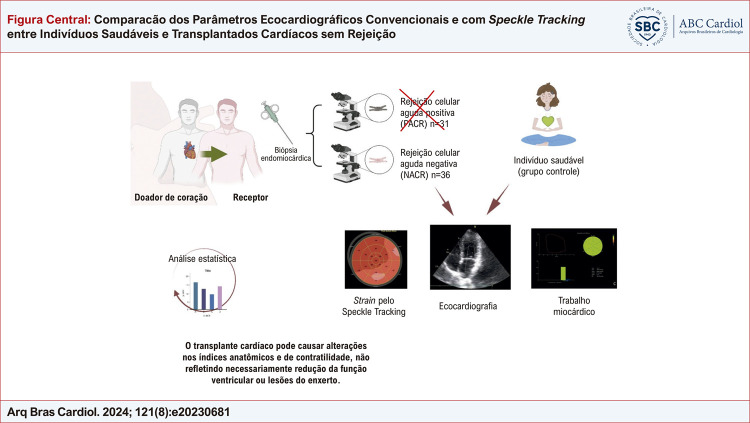
: Comparacão dos Parâmetros Ecocardiográficos Convencionais e com *Speckle Tracking* entre Indivíduos Saudáveis e Transplantados Cardíacos sem Rejeição

### Ecocardiograma transtorácico

As imagens foram capturadas por ETT realizado no mesmo dia da BEM, com intervalo inferior a 4 horas entre o exame de imagem e a biópsia, por três médicos cardiologistas treinados, em um aparelho de ultrassom comercialmente disponível com transdutor de 5 MHz (GE Vivid 9, GE Healthcare, Milwaukee, Wisconsin, USA) e todas as medidas foram realizadas *off-line* em uma estação de trabalho no *software EchoPAC Versão 202 (GE Vingmed Ultrasound, Norway)* .

Os cardiologistas envolvidos na análise não tiveram acesso ao resultado da biopsia até que todos os parâmetros fossem analisados.

Imagens ecocardiográficas padrão foram adquiridas de acordo com a Sociedade Americana de Ecocardiografia ( *American Society of Echocardiography* – ASE).^2^ A FEVE foi obtida pelo método de Simpson nas janelas apical 4 e 2 câmaras e a massa ventricular esquerda foi calculada usando a equação proposta por Devereux e indexada pela superfície corpórea. A função ventricular direita foi avaliada com os parâmetros convencionais recomendados pela diretriz de avaliação do ventrículo direito (VD):^2^ excursão sistólica do anel tricúspide (TAPSE), velocidade de excursão do Doppler tecidual do anel tricúspide (S do VD) e fração de encurtamento (FAC).

A função diastólica foi avaliada por meio das velocidades de influxo mitral (ondas E e A), razão E/A, velocidade do Doppler tecidual mitral (e’/a’) e relação E/e’.^10^

O *Strain* miocárdico do ventrículo esquerdo (VE) foi obtido pelo *Strain* global longitudinal pelo método do *Speckle Tracking* 2D^11^ e expresso em valor absoluto conforme atuais recomendações.^12^

**Figura 1:**
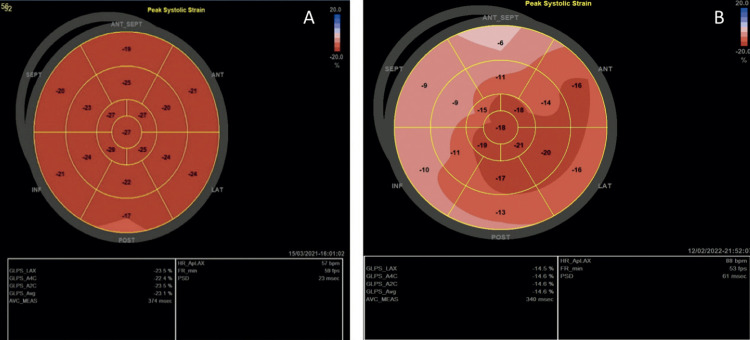
– Comparação de Strain Global Longitudinal do Ventrículo Esquerdo entre indivíduo do grupo controle e transplantado cardíaco. Mapa Polar dos 17 segmentos e resultado das médias de cada janela apical. A: Indivíduo normal, B Transplantado Cardíaco.

Nesta análise foram obtidos 3 ciclos cardíacos consecutivos em cada plano apical (apical 4 câmaras, 3 câmaras e 2 câmaras), com taxa de quadros acima de 50 por segundo. Essas imagens foram transferidas para a estação de trabalho e analisadas no *software EchoPAC Versão 2.02 (GE Vingmed Ultrasound, Norway)* . As bordas endocárdicas foram manualmente traçadas no final da sístole no ciclo cardíaco, começando na janela apical longitudinal, onde é mais simples de identificar o tempo de fechamento da valva aórtica. O *software* utiliza uma região de interesse (ROI) de toda a espessura miocárdica, a qual pode ser manualmente ajustada em largura, se necessário, e uma imagem em movimento exibe o rastreamento. O *software* então divide o VE em seis segmentos, calculando *strain* global longitudinal e segmentar. O mesmo processo foi repetido para o apical 4 e 2 câmaras e o SGL VE foi determinado pela média dos valores locais de todos os segmentos miocárdicos e exibido em formato de mapa polar ( Figura 1 ).

Fonte: A Autora, 2022.

*Strain* do Ventrículo Direito (SL VD) foi analisado por meio do *Strain* Longitudinal da parede livre, com a obtenção de três ciclos cardíacos na janela apical 4 câmaras focada no VD^13^ e de marcadores temporais de abertura e fechamento da valva pulmonar obtidas pelo Doppler contínuo ao nível da valva pulmonar. A média dos 3 segmentos da parede livre do ventrículo direito: basal, medial e apical foi considerado o SL VD.

Usando as mesmas imagens do SGL VE e obtendo a pressão arterial sistólica e diastólica, obteve-se o Índice de Trabalho Miocárdico (ITM).^14^

**Figura 2:**
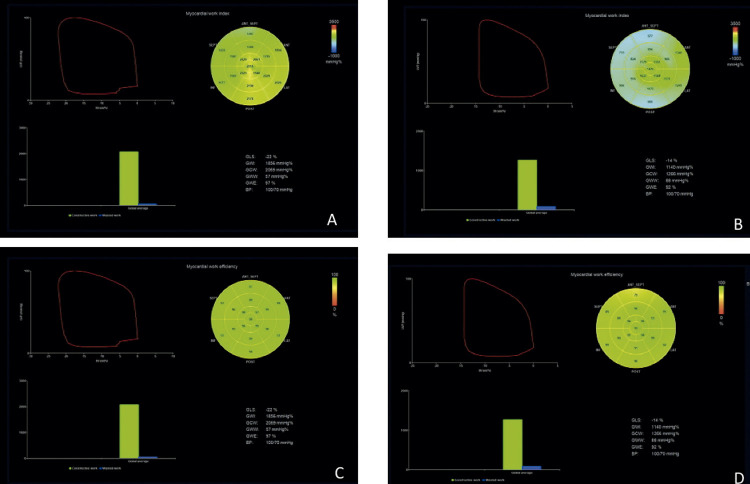
– Comparação dos Índices de Trabalho Miocárdico do Ventrículo Esquerdo entre transplantado cardíaco e indivíduo saudável. Mapa Polar dos 17 segmentos (A: ITM indivíduo normal, B: ITM Transplantado Cardíaco, C: TEG Indivíduo normal e D: TGD Transplantado Cardíaco), gráfico demonstrando Strain/curva de pressão total e gráfico com barras do trabalho desperdiçado (azul) e trabalho construtivo (verde). ITM: índice de trabalho miocárdico; TEG: trabalho de eficiência global; TGD: trabalho global desperdiçado.

O *software* incorporou a pressão ventricular esquerda não invasiva estimada por um manguito automático ao *strain* do VE, fornecendo os índices associados a curva de *strain* -pressão ( Figura 2 ). Ao longo de valores segmentar e global para o ITM, índices adicionais foram fornecidos de acordo com estudo NORRE:^4^

Fonte: A Autores, 2022.

- Índice de Trabalho Miocárdico (ITM): é o trabalho total dentro da área de pressão/ strain longitudinal, calculado do fechamento a abertura da valva mitral.
- Trabalho Construtivo Global (TCG): é o trabalho realizado durante encurtamento na sístole adicionando trabalho negativo durante o estiramento na fase isovolumétrica de relaxamento.
- Trabalho Global Desperdiçado (TGD): é o trabalho negativo realizado durante o estiramento na sístole adicionando o trabalho durante o encurtamento no relaxamento isovolumétrico. É o oposto do que é esperado no tempo do ciclo cardíaco e não contribui para a ejeção ventricular esquerda.
- Trabalho de Eficiência Global (TEG): é o trabalho construtivo dividido pela soma do trabalho construtivo e desperdiçado, reportada em porcentagem (0–100%).

Os prontuários médicos eletrônicos de todos os participantes foram revisados no dia dos exames (BEM e ETT) para comparação clínica, demográfica e esquema de drogas imunossupressoras. Características clínicas e a lista de drogas imunossupressoras foram coletadas.

### Análise estatística

As variáveis contínuas foram apresentadas como média ± Desvio Padrão – DP ou mediana ± amplitude interquartil/ intervalo interquartil e as variáveis categóricas foram apresentadas como frequência (percentual do total). Os dados contínuos foram analisados com teste t de Student não pareado ou Mann-Whitney quando os pressupostos de normalidade não foram atendidos pelo teste de normalidade de Shapiro-Wilk. As variáveis categóricas foram analisadas usando o teste χ2 de Pearson.

As análises foram conduzidas pelo software SAS 9.4 e p < 0,05 foi considerado significativo para análises gerais.

A variabilidade interobservador foi realizada comparando as medidas de 20 pacientes obtidas por três diferentes ecocardiografistas treinados e a variabilidade intraobservador foi realizada 1 mês após as medidas por um investigador.

## Resultados

**Figura 3:**
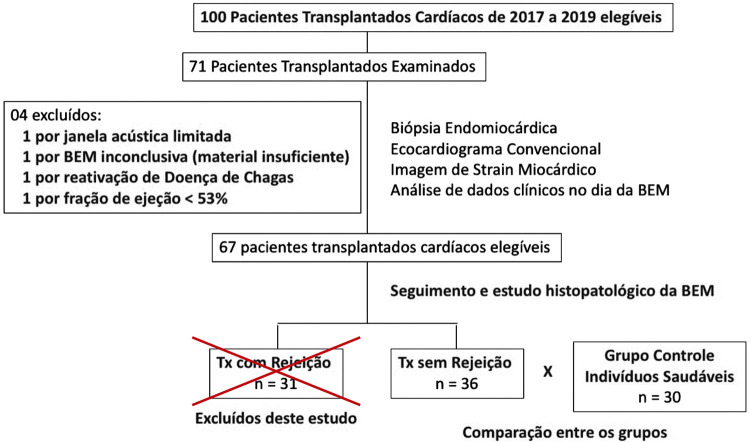
– Fluxograma dos pacientes Transplantados Cardíacos no período de 2017 a 2019. BEM: biópsia endomiocárdica; TX: transplantados.

Dos 100 pacientes transplantados na Instituição no período, 71 foram incluídos, totalizando 120 biópsias, com intervalo mínimo de 6 dias, máximo de 328 dias e mediana de 70 dias. Entretanto, de acordo com os critérios de exclusão, 35 pacientes foram excluídos (1 por apresentar janela acústica limitada, 1 por BEM inconclusiva, 1 por reativação da Doença de Chagas, 1 por fração de ejeção menor que 53% e 31 pacientes por apresentarem BEM com rejeição), restando 36 pacientes transplantados para análise do estudo, sendo comparados com 30 indivíduos saudáveis no grupo controle ( Figura 3 ).

Fonte: A Autora, 2022.

Na análise das características dos grupos, observou-se que a idade e o gênero não diferiram entre os transplantados e o grupo controle pelo teste de qui-quadrado. O grupo transplantado sem rejeição apresentou a medida da pressão arterial e frequência cardíaca mais alta e o grupo controle esteve com maior medida de peso, consequentemente, maior índice de massa corporal ( Tabela 1 ).

**Tabela 1:** – Características clínicas dos grupos transplantados cardíacos sem rejeição e indivíduos saudáveis (controle)

Característica Clínica	Transplantados sem Rejeição (n=36)				Controle (n=30)				p-valor

	Média ou Mediana	DP/QR	QT 1	QT 3	Média ou Mediana	DP/ QR	QT 1	QT 3	
**Idade (anos)**	52,50	12,50	44,00	56,50	46,00	7,00	44,00	51,00	0,2450^†^
**Gênero feminino**	17 (40,48%)	-	-	-	25 (59,52%)	-	-	-	0,2826^‡^
**PAS (mmHg)**	122,00	31,00	111,00	142,00	117,00	17,00	111	128	0,1910^†^
**PAD (mmHg)**	83,33	16,94	-	-	69,80	10,15	-	-	0,0002^*^
**FC (bpm)**	83,61	13,97	-	-	75,17	9,55	-	-	0,0051^*^
**Altura (cm)**	164,00	13,00	158,00	171,00	165,50	17,00	158	175	0,5706^†^
**Peso (kg)**	60,75	10,01	-	-	72.86	11,87	-	-	<0,0001^*^
**SC (m^2^)**	1,66	0,15	-	-	1,80	0,22	-	-	0,0046^*^
**IMC (cm^2^/kg)**	21,59	3,86	20,26	24,12	26,18	2,92	20,26	24,12	<0,0001^†^

* p-valor calculado pelo teste t de Student. † p-valor calculado pelo teste não paramétrico de Mann-Whitney. ‡ p-valor calculado pelo teste de Qui-quadrado. QR: Amplitude interquartil; PAS: pressão arterial sistólica; PAD: pressão arterial diastólica; FC: frequência cardíaca; SC: superfície corpórea; IMC: índice de massa corporal; QT: quartil. Obs: valores expresso em média e desvio-padrão, ou mediana e amplitude interquartil/ com os intervalor do primeiro e terceiro quartil, ou frequência (%).

Dentre os pacientes transplantados, 19 (52,78%) apresentaram doador do mesmo gênero que o receptor, a maioria dos doadores foram do gênero masculino (63%). Com relação a droga imunossupressora, 26 estavam em uso de tacrolimus (72,22%), 10 em uso de ciclosporina (27,78%), 25 em uso de micofelonato (69,44%), 11 em uso de azatioprina (30,56%) e 34 em uso de prednisona (94,44%) e quanto a presença de comorbidades, 8 transplantados (22,22%) eram portadores de hipertensão arterial e 9 (25%) eram diabéticos, sendo a Doença de Chagas, a etiologia mais frequente de insuficiência cardíaca pré-transplante, com 52,77%. O tempo entre o TC e a realização da BEM e ecocardiograma apresentou uma mediana de 70 dias com uma amplitude interquartil de 63 dias ( Tabela 2 ).

**Tabela 2:** – Características clínicas dos transplantados cardíacos sem rejeição

Variáveis	N
**Intervalo entre o transplante cardíaco e a biópsia (dias)**	70
Quartil 0	7
Quartil 1	11
Quartil 2	70
Quartil 3	169
**Drogas Imunossupressoras**	
Tacrolimus	26 (72,22%)
Ciclosporina	10 (27,78%)
Micofelonato	25 (69,44%)
Azatioprina	11 (30,56%)
Prednisona	34 (94,44%)
**Características clínicas**	
Doador mesmo gênero do receptor	19 (52,78%)
DM	9 (25,00%)
Hipertensão	8 (22,22%)
**Etiologia da Insuficiência Cardíaca**	
Cardiotoxicidade	1 (2,77%)
Miocardio não compactado	1 (2,77%)
Miocardiopatia Chagásica	19 (52,77%)
Miocardiopatia Idiopática	6 (16,66%)
Miocardiopatia Isquêmica	3 (8,33%)
Miocardiopatia Periparto	2 (5,55%)
Miocardiopatia Restritiva	1 (2,77%)
Miocardiopatia valvar	2 (5,55%)
Tetralogia de Fallot	1 (2,77%)

DM: dabetes mellitus.

Com relação às medidas ecocardiográficas, os pacientes transplantados sem rejeição apresentaram uma medida maior do septo interventricular e da parede posterior, consequentemente maior espessura relativa (ER) e massa ventricular. Observou-se maior volume do átrio esquerdo, com média de 38,17 ml/m^2^ e menor valor nas medidas de Doppler tecidual em relação ao grupo controle. Entretanto, as medidas que envolvem a função diastólica não puderam ser analisadas em mais de 50% dos transplantados devido a problemas técnicos (fusão de ondas), com possível perda de confiabilidade dos dados ( Tabela 3 ).

**Tabela 3:** – Comparação entre as variáveis ecocardiográficas de transplantados cardíacos sem rejeição e voluntários saudáveis (controle)

Variável Ecocardiográfica*	Transplantados sem Rejeição (n=36)				Controle (n=30)				p-valor

	Média ou Mediana	DP ou QR	QT 1	QT 3	Média ou Mediana	DP ou QR	QT 1	QT 3	
EDS (mm)	10,00	10,00	9,00	11,50	8,00	1,00	7,00	8,00	<0,0001^‡^
EDPP (mm)	9,50	9,50	8,00	11,00	7,50	1,00	7,00	8,00	<0,0001^‡^
DDVE (mm)	43,69	3,09	-	-	44,70	3,20	-	-	0,2011^†^
DSVE (mm)	28,00	28,00	26,50	31,00	28,00	3,00	26,00	29,00	0,5027^‡^
FE Teicholz (%)	65,49	5,23	62,53	67,76	66,91	6,35	64,43	70,78	0,0491^‡^
VDFVE Simpson (mL)	74,99	23,00	60,00	83,00	73,00	28,00	63,00	91,00	0,9790^‡^
VSFVE Simpson (mL)	26,00	16,00	21,00	37,00	27,00	13,00	20,00	33,00	0,8075^‡^
FE Simpson (%)	64,52	6,88	59,78	66,67	62,90	6,74	60,29	67,03	0,7371^†^
Massa Devereux (g)	148,69	44,51	-	-	108,15	25,88	-	-	<0,0001^†^
Indice Massa (g/m^2^)	90,79	29,89	-	-	59,91	11,53	-	-	<0,0001^†^
Espessura Relativa	0,44	0,09	-	-	0,33	0,04	-	-	<0,0001^†^
Volume AE (mL)	61,75	29,50	49,75	79,25	34,50	12,00	30,00	42,00	<0,0001^‡^
Volume AEi (mL/ m^2^)	38,17	12,90	31,21	44,11	18,98	7,18	16,29	23,47	<0,0001^‡^
Volume AD (mL)	35,00	19,00	25,00	44,00	27,50	12,00	22,00	34,00	0,0200^‡^
Volume Adi (mL/ m^2^)	19,91	11,62	15,40	27,02	15,61	7,51	11,55	19,07	0,0014^‡^
VD basal (mm)	33,64	5,79	-	-	33,93	3,58	-	-	0,8014^†^
VD médio (mm)	29,11	5,41	-	-	26,33	3,96	-	-	0,0226^†^
S VD (cm/s)	7,40	2,06	-	-	13,37	2,08	-	-	<0,0001^†^
TAPSE (mm)	12,51	3,18	-	-	22,97	3,01	-	-	<0,0001^†^
FAC VD (%)	42,85	7,51	-	-	44,37	4,55	-	-	0,3159^†^

* valores expresso em média e desvio-padrão, ou mediana e amplitude interquartil. † p-valor calculado pelo teste t de Student. ‡ p-valor calculado pelo teste não paramétrico de Mann-Whitney. QR: Amplitude Interquartil; EDS: espessura do septo do ventrículo esquerdo; EDPP: espessura da parede posterior; DDVE: diâmetro diastólico do ventrículo esquerdo; DSVE: diâmetro sistólico do ventrículo esquerdo; FE: fração de ejeção; VDFVE: volume diastólico final do ventrículo esquerdo; VSFVE: volume sistólico final do ventrículo esquerdo; AE: átrio esquerdo; AEi: átrio esquerdo indexado pela superfície corpórea; AD: átrio direito; ADi: átrio direito indexado pela superfície corpórea; VE: ventrículo esquerdo; VD: ventrículo direito; TAPSE: excursão sistólica do anel tricúspide; FAC: fração de encurtamento; QT: quartil.

Na avaliação da função ventricular direita, observou-se S de VD e TAPSE com valores inferiores para os transplantados sem rejeição, porém com FAC dentro da normalidade ( Tabela 3 ).

Observou-se redução significativa dos valores do *Strain* pelo *Speckle Tracking* na comparação entre os grupos, abaixo dos valores de referência^2^ nos pacientes transplantados, tanto para o SGL VE, quanto para o SL VD ( Tabela 4 ).

**Tabela 4:** – Comparação do *Strain* pelo *Speckle Tracking* e Trabalho Miocárdico entre os pacientes transplantados sem rejeição e indivíduos saudáveis (grupo controle)

*Strain* / Trabalho Miocárdico^*^	Transplantados sem Rejeição (n=36)				Controle (n=30)				p-valor

	Média ou Mediana	DP ou QR	QT 1	QT 3	Média ou Mediana	DP ou QR	QT1	QT3	
SGL VE	11,99	2,740	-	-	20,60	2,15	-	-	<0,0001^†^
SL VD	16,67	4,33	18,00	13,67	25,50	7,03	30,33	23,30	<0,0001^‡^
ITM (mmHg %)	1131,69	469,43	-	-	2005,10	339,10	-	-	<0,0001
TEG (%)	85,43	8,70	-	-	96,30	2,07	-	-	<0,0001
TCG (mmHg %)	1395,91	505,62	-	-	2758,50	3529,20	-	-	<0,0001
TGD (mmHg %)	182,11	143,90	-	-	68,55	52,58	-	-	<0,0001

* valores expresso em média e desvio-padrão, ou mediana e amplitude interquartil. † p-valor calculado pelo teste t de Student. † p-valor calculado pelo teste não paramétrico de Mann-Whitney. SGL VE: Strain Global Longitudinal do Ventrículo Esquerdo; Strain VD: Strain parede livre do Ventrículo Direito; ITM: índice de trabalho miocárdico; TEG: trabalho de eficiência global; TCG: trabalho construtivo global; TGD: trabalho global desperdiçado; QT: quartil.

Os índices de trabalho miocárdico encontraram-se reduzidos em pacientes transplantados sem rejeição em comparação ao grupo controle ( tabela 4 ). O valor médio das variáveis ITM, TEG e TCG são significativamente menores para transplantados em relação aos indivíduos saudáveis (p < 0,0001), sendo o TGD maior no grupo de transplantados sem rejeição.

O coeficiente de correlação intraclasse do SGL VE foi 0,98 (95% IC =0,94-0,99) para o coeficiente de variabilidade interobservador e 0,88 (95% IC =0,70-0,99) para a variabilidade intraobservador. Os coeficientes de correlação intraclasse do SL VD foram 0,97 (95% IC =0,94-0,95) para a variabilidade interobservador e 0,98 (95% IC =0,95-0,99) para a variabilidade intraobservador. As variáveis interobservador do Trabalho Miocárdico derivado do SGL estão descritas: ITM 0,93 (95% IC =0,84-0,97), TEG 0,97 (95% IC= 0,93-0,99), TGC 0,94 (95% IC=0,85-0,97) e TGD 0,92 (95% IC=0,81-0,97).

## Discussão

O principal achado do presente estudo é a observação de valores de mecânica cardíaca, obtidos por meio do *Strain* pelo *Speckle Tracking* e do trabalho miocárdico, reduzidos em uma coorte de pacientes transplantados sem rejeição em comparação com indivíduos saudáveis com idade e gênero semelhantes. Encontrou-se, para os transplantados, redução nos valores de *Strain* Global Longitudinal de VE, *Strain* de parede livre de VD e dos índices de trabalho miocárdico, maior tamanho do átrio esquerdo, maior índice de massa e espessura relativa das paredes e a presença da Doença de Chagas como principal etiologia de insuficiência cardíaca que culminou no TC.

Em concordância com estudos envolvendo transplantados cardíacos,^5^ os dados deste estudo constataram remodelamento ventricular com aumento do índice de massa de VE e da ER. Badano et al.^15^ descreveram que durante os primeiros meses após o TC pode ocorrer aumento da massa ventricular esquerda e da espessura da parede, provavelmente causada por infiltração inflamatória celular e edema do enxerto. Durante o seguimento, um aumento secundário da massa do VE e da espessura da parede pode ocorrer como consequência de múltiplos fatores como episódios repetidos de rejeição (ausente nos pacientes participantes deste estudo), taquicardia crônica, injuria isquêmica e hipertensão sistêmica, usualmente induzida por agentes imunossupressores.^16^

No grupo transplantado, a fração de ejeção se manteve dentro da normalidade (FEVE 64,52±6,88%), porém os índices de *Strain* pelo *Speckle Tracking* encontraram-se reduzidos (SGL VE 11,99±2,74) em comparação ao grupo controle e aos padrões de normalidade descritos em diretrizes.^2^

Estudos relataram que medidas de *strain* estão reduzidas em muitas situações clínicas com fração de ejeção preservada,^17 , 18^ no entanto, ainda é incerto o significado clínico dos valores abaixo da normalidade destes parâmetros no primeiro ano do transplantado cardíaco. Badano et al.^15^sugerem a análise após seis meses do ecocardiograma no TC para determinar os valores basais de normalidade do coração transplantado, quando a injúria isquêmica é menor.

Os achados deste estudo foram concordantes com outras publicações que observaram valores de SGL reduzidos após o transplante, mesmo em pacientes sem rejeição.^5 , 15^ O procedimento cirúrgico e o remodelamento cardíaco, além da inflamação inicial, podem contribuir para a queda do SGL. Ingvarsson et al.^5^ descreveram como hipótese para valores baixos de *strain* uma possível insuficiência cardíaca com fração de ejeção preservada em grupo de transplantados cardíacos, visto apresentarem alterações em massa ventricular e padrão restritivo de disfunção diastólica.

Apesar da superioridade do SGL sobre a FEVE em avaliações da performance sistólica do VE,^14^ esta técnica é ainda limitada devido a dependência do enchimento ventricular afetando a avaliação da função contrátil do miocárdio em condições específicas. Para minimizar os efeitos da pós-carga da pressão arterial no *strain* , o trabalho miocárdico surge como nova ferramenta ecocardiográfica que permite quantificar a performance ventricular esquerda baseada na curva de *strain* /pressão. Este estudo é o primeiro na literatura a descrever o trabalho miocárdico em transplantados cardíacos.

Observou-se redução dos índices de trabalho miocárdico e aumento do trabalho desperdiçado no grupo de transplantados. Estes dados corroboraram que o comportamento do coração transplantado, mesmo sem rejeição, apresenta uma performance reduzida com maior trabalho desperdiçado, tendo como hipótese alterações na dinâmica cardíaca já descritas após a cirurgia ou até mesmo pelo quadro inicial de inflamação.^15^

Os índices que avaliaram a função ventricular direita, tais como S do VD, TAPSE e *Strain* da parede livre, encontraram-se reduzidos nos transplantados em comparação ao grupo controle (p<0,0001) e a fração de encurtamento e tamanho ventricular permaneceram normais.

Estudos prévios obtiveram resultados semelhantes,^5^ sendo importante mencionar que o TAPSE e S do VD são ineficazes em diferenciar a contração ativa da contração passiva causada pelo VE e por isso não refletem a função do VD verdadeira. Raina et al.^19^ sugeriram que a cirurgia torácica altera a contratilidade do ventrículo direito, diminuindo a contração longitudinal e aumentando a contração radial. Este achado foi confirmado em estudo experimental usando os cérebros de doadores, em que o aumento da pressão intracraniana e dos níveis de catecolaminas estão relacionados a redução da função ventricular direita após o TC.^20^ Dessa forma, foi constatado que a qualidade da manutenção do doador, técnica do explante, tempo de isquemia, hipotermia e implante no receptor têm impacto na função do ventrículo direito após o transplante.^5^

Neste estudo, observou-se um aumento significativo do tamanho atrial esquerdo, apesar da técnica cirúrgica bicaval ter sido utilizada na totalidade dos pacientes transplantados, em contraste com a técnica biatrial, mais antiga e com característica de proporcionar átrios maiores. A presença de átrios maiores, apesar da utilização da técnica bicaval, pode indicar uma desproporção entre o tamanho do enxerto do doador em relação ao receptor, resultando em um volume indexado pela superfície corpórea maior ou até mesmo um endurecimento diastólico progressivo com aumento das pressões de enchimento causando disfunção diastólica e aumento atrial.^1^

A população estudada, diferentemente de outros estudos relatados, tem como causa do transplante a Doença de Chagas (acima de 50%). No entanto, no grupo de pacientes estudados, não se constatou reativações desta doença e, pelo número reduzido de pacientes, não foi possível avaliar diferenças nos parâmetros de ecocardiograma convencional ou na mecânica cardíaca relacionado a presença dessa patologia em relação às demais causas de insuficiência cardíaca, fato comprovado também por Otto et al.^7^

O presente estudo apresenta algumas limitações. Este foi um estudo com único centro, não se obteve informações sobre as características do doador pois muitos órgãos são de região remota ao centro pesquisador, apresentou um número reduzido de pacientes e utilizou apenas um tipo de Software para a avaliação.

A técnica do *Strain* pelo *Speckle Tracking* e o trabalho miocárdico são ferramentas em evolução e os valores obtidos neste estudo não podem ser aplicados a outros *softwares* para análise de *strain* . Um estudo multicêntrico incluindo diversos equipamentos de ecocardiograma e um número maior de pacientes deve ser realizado.

No entanto, como ponto forte do estudo, vale ressaltar que este é o primeiro a avaliar o trabalho miocárdico em pacientes transplantados sem rejeição e observar a mecânica cardíaca distinta da observada em um grupo comparável de indivíduos saudáveis.

## Conclusão

No primeiro ano de evolução, pacientes transplantados cardíacos sem rejeição comprovada por BEM apresentaram *Strain* Global Longitudinal do VE, *Strain* da parede livre do ventrículo direito e trabalho miocárdico reduzidos, mas fração de ejeção semelhante em relação a um grupo comparável de indivíduos saudáveis. Nos pacientes transplantados observou-se adicionalmente aumento do átrio esquerdo e maior remodelamento do VE.

Estes achados indicam que o padrão dos parâmetros de ecocardiograma difere entre transplantados cardíacos sem rejeição e indivíduos saudáveis, o que pode ser clinicamente relevante no seguimento desses pacientes.

Vale destacar que para a determinação de valores de referência das medidas ecocardiográficas específicas para transplantados é necessário a realização de estudos multicêntricos e com um número maior de pacientes.
