# Supplementary material for: Comparacão dos Parâmetros Ecocardiográficos Convencionais e com Speckle Tracking entre Indivíduos Saudáveis e Transplantados Cardíacos sem Rejeição
Source: Arq Bras Cardiol. 2024 Jul 25;121(8):e20230681. [Article in Portuguese] doi: 10.36660/abc.20230681 (PMC11463340; doi:10.36660/abc.20230681)
Supplement: Translation

# Translation: Comparative Analysis of Conventional and Speckle Tracking Echocardiographic Variables between Patients with Unrejected Heart Transplants and Healthy Individuals

**DOI:** 10.36660/abc.20230681i

**Authors:** Aline Oliveira Martins Campo Dall’Orto, Maria Estefania Otto, Simone Ferreira Leite, Marco Antônio Freitas de Queiroz Maurício, Natália Taveira Martins, Samuel Rabelo Araújo, Soraya Vasconcelos Almeida, Luiza Valle Oliveira Brizida, Fernando Antibas Atik

**Affiliations:** 1 Brazil Universidade de Brasilia, Brasília, DF – Brazil; 2 Brazil Instituto de Cardiologia e Transplantes do Distrito Federal (ICTDF), Brasília, DF – Brazil

**Keywords:** Heart Transplantation, Echocardiography, Ventricular Function

## Abstract

**Background:**

Echocardiography is essential for the assessment of patients with heart transplants. However, normal values in such individuals are not clearly defined.

**Objectives:**

To compare conventional echocardiographic and speckle tracking variables between patients with unrejected heart transplants and healthy individuals.

**Methods:**

:A prospective study was conducted with adult patients having undergone heart transplantation at least one year earlier and submitted to endomyocardial biopsy followed by transthoracic echocardiogram (TTE). Conventional TTE measures and mechanical heart strain assessments using speckle tracking were performed and the results were compared to those of a group of healthy volunteers. Statistical significance was set at 5% (p < 0.05).

**Results:**

Thirty-six transplant patients without rejection were analyzed and compared to 30 healthy individuals. Chagas disease was the main reason for transplantation. Lower left ventricular global longitudinal strain expressed in absolute values was found (11.99% in transplant patients vs. 20.60% in controls; p <0.0001), right ventricular free wall longitudinal strain (16.67% in transplant patients vs. 25.50% in controls; p <0.0001) and myocardial work indices (p < 0.0001) as well as a larger size of the left atrium (38.17 ml/m2 in transplant patients vs. 18.98 ml/m2 in controls; p <0.0001) and greater mass and relative wall thickness (p <0.0001).

**Conclusion:**

Stable patients having undergone heart transplants without rejection have differences concerning echocardiographic variables compared to healthy individuals. These findings indicate that conventional echocardiographic measures and heart mechanics are altered in transplant patients even in the absence of rejection. Such findings are relevant to the clinical context and follow-up of the patient.

**Central Illustration:** : Comparative Analysis of Conventional and Speckle Tracking Echocardiographic Variables between Patients with Unrejected Heart Transplants and Healthy Individuals

## Introduction

Echocardiography is a useful tool that is easy to perform for the assessment of patients with heart transplants in different stages of the transplantation process – from the intraoperative procedure to the postoperative follow-up assessments. This technique is also important to the monitoring of complications following endomyocardial biopsies and acute cellular rejection as well as the diagnosis of vascular disease of the graft.^1^

The normalization of left ventricular function after transplantation leads to an improvement in symptoms and exerts a considerable impact on the prognosis.^1^Ejection fraction assessed by an echocardiogram is used as a measure of ventricular function.^2^

A novel two-dimensional echocardiogram modality denominated speckle tracking enables the detection of left ventricular dysfunction before a change in the ejection fraction through the analysis of myocardial deformation (strain). Adding the determination of blood pressure to this measure enables the determination of myocardial work. This novel variable is superior to longitudinal global strain in the assessment of oxygen demand and heart function, as it considers myocardial deformation together with the afterload.^3^

In normal individuals, changes in ventricular function are well described through normality values compiled in various studies.^2 , 4^For patients having undergone heart transplantation, however, information on the normal performance of the graft remains scarce due to limited scientific evidence in available studies.^5^

Therefore, the present study aimed to compare echocardiographic variables between patients having undergone heart transplantation without rejection and a group of healthy individuals, with an investigation of mean left ventricular longitudinal global strain (LV GLS) and right ventricular free wall longitudinal strain (RV FWLS) and well as myocardial work indices in the two groups.

## Methods

### Population

The sample size was calculated based on studies that compared LV GLS in transplant patients,^6 , 7^adopting an 80% power to detect a 10% reduction in LV GLS, considering 21% (normal absolute value) with a 4% standard deviation and 5% alpha error. The complete description of the sample calculation can be found in a previous study published by the authors comparing patients with and without rejection, for which 34 patients were needed in each group.^7^For the comparison of patients without rejection and healthy controls, however, 25 patients were determined necessary for each group. The present study involved 36 patients without rejection and 30 healthy individuals (numbers higher than 25).

Adults (18 years of age or older) were included. In the heart transplant group, patients having undergone transplantation at least one year earlier were prospectively studied at a reference hospital between January 2017 and December 2019. A group of healthy volunteers without comorbidities who agreed to participate in the study were included in the control group. These volunteers were submitted to basic laboratory exams, an electrocardiogram, and a noninvasive blood pressure measurement before the echocardiogram.

The exclusion criteria for the transplant patients were left ventricular ejection fraction (LVEF) below 53%, relapse of Chagas disease, acute cell rejection identified by biopsy at some point during follow-up, humoral rejection, irregular heart rate, confirmed vascular disease of graft, limited acoustic window and refusal to participate.

This study received approval from the Human Research Ethics Committee. Its inscription number on the Brazil Platform is 65910517.0.0000.0026. All participants signed a statement of informed consent.

### Endomyocardial biopsy

The transplant patients were in routine follow-up, during which vigilance endomyocardial biopsy (EMB) was performed, followed by a transthoracic echocardiogram on the same day, with less than four hours between exams. The vigilance EMB protocol consists of a biopsy once a week in the first month, every two weeks in the second month, and monthly from three to seven months after the transplant, totaling nine biopsies in six months. After seven months, myocardial scintigraphy is executed and biopsy is only performed when the result is positive for inflammation.

EMB was performed by fluoroscopy through the femoral vein, with three to five fragments collected in the region of the interventricular septum accessed by the right ventricle. The samples were analyzed under an optical microscope after fixation in hematoxylin and eosin.^8^The pathologist in charge of the biopsy analysis was unaware of the echocardiographic results. The findings were classified based on the grading system for the assessment of rejection proposed by the International Society for Heart and Lung Transplantation in 2004.^9^

The transplant patients were divided into two groups based on the histopathological result of the EMB: Group 1 – without acute cellular rejection (Grades 0 and 1R) and Group 2 – with acute cellular rejection (Grades: 2R and 3R). The patients in Group 2 were excluded from the analysis. The patients in Group 1 (heart transplant without rejection) were compared to the healthy individuals who composed the control group (central figure).

### Transthoracic echocardiogram

Images were captured by TTE on the same day as the EMB, with less than four hours between the biopsy and imaging exam, by three trained cardiologists using a commercially available ultrasound device with a 5-MHz transducer (GE Vivid 9, GE Healthcare, Milwaukee, Wisconsin, USA). All measurements were made offline at a workstation using the EchoPAC program, version 202 (GE Vingmed Ultrasound, Norway *)* . The cardiologists had no access to the biopsy results until all variables of interest had been analyzed.

Standard echocardiographic images were acquired as recommended by the American Society of Echocardiography.^2^LVEF was calculated using the Simpson method in four-chamber and two-chamber apical views. LV mass was calculated using the equations proposed by Devereux et al.^2^and indexed by body surface. RV function was assessed using the conventional variables recommended by RV assessment guidelines:^2^tricuspid annular plane systolic excursion (TAPSE), tricuspid annular systolic velocity of tissue Doppler (RV S), and fractional area change (FAC). Diastolic function was assessed based on mitral inflow velocities (E and A waves), E/A ratio, mitral tissue Doppler velocity (e’/a’), and E/e’ ratio.^10^

LV myocardial strain was obtained by global longitudinal strain (GLS) using 2D speckle tracking^11^and expressed in absolute values, following current recommendations.^12^In this analysis, three consecutive heart cycles were obtained on each apical plane (four chambers, three chambers, and two chambers), with a frame rate higher than 50 per second. These images were transferred to the workstation and analyzed using the EchoPAC program, version 2.02 (GE Vingmed Ultrasound, Norway). The endocardial edges were manually traced at the end of the systole of the heart cycles, beginning in the longitudinal apical view, where it is easier to identify the aortic valve closure time. The software program uses a region of interest (ROI) of the entire myocardial thickness, which can be manually adjusted in width, if necessary, and a moving image exhibits the tracking. The software program then divides the left ventricle into six segments, calculating global and segmental longitudinal strain. The same process was repeated for the four-chamber and two-chamber apical views. The LV GLS was determined by the mean of the local values of all myocardial segments and exhibited in a polar map format ( Figure 1 ).

**Figure 1:** – Comparison of Global Longitudinal Strain of the Left Ventricle between individuals in the control group and heart transplant recipients. Global longitudinal strain polar map from 17 segments. A: Normal individual, B Heart Transplant without rejection.

Right ventricle longitudinal strain (RV FWLS) was analyzed by the longitudinal strain of the free wall, with the obtainment of three heart cycles in the four-change apical view focused on the RV^13^and temporal opening and closure markers of the pulmonary valve obtained by continuous Doppler at valve level. The mean of the three segments of the free wall of the RV (basal, medial, and apical) was considered the RV FWLS.

The myocardial work index (MWI) was obtained using the same images of the LV GLS and determining systolic and diastolic blood pressure.^14^The software program incorporated LV pressure estimated noninvasively by an automatic cuff at the strain of the LV, providing indices associated with the strain-pressure curve ( Figure 2 ). Throughout the segmental and global values for the MWI, additional indices were obtained based on the NORRE study:^4^

**Figure 2:** – Comparison of Left Ventricular Myocardial Work Indices between heart transplant recipients and healthy individuals. Polar Map from 17 segments (A: MWI normal individual, B: MWI Heart Transplant, C: GEW Normal individual, and D: GWW Heart Transplant), graph demonstrating Strain/total pressure curve and bar graph of wasted work (blue) and constructive work (green). MWI: Myocardial Work Index; GEW: Global Efficiency Work; GWW: Global Wasted Work.

- Myocardial work index (MWI) is the total work within the pressure/longitudinal strain area calculated from closure to opening of the mitral valve.
- Global constructive work (GCW) is the sum of positive work due to myocardial shortening during systole and negative work due to lengthening during isovolumic relaxation.
- Global wasted work (GWW) is the sum of negative work during myocardial lengthening in systole and positive work during shortening in isovolumic relaxation. GWW is the opposite of what is expected at the time of the heart cycle and does not contribute to LV ejection.
- Global work efficiency (GWE) was derived from the percentage ratio of constructive work to the sum of constructive work and wasted work expressed in percentage (0–100%).

The electronic medical records of all participants were reviewed on the day of the exams (EMB and TTE) for the comparison of clinical and demographic characteristics and the immunosuppressant regimen. Clinical characteristics and the lists of immunosuppressants were collected.

### Statistical analysis

Continuous variables were expressed as mean and standard deviation (SD) or median and interquartile range. Categorical variables were expressed as frequency (percentage of total). The Shapiro-Wilk test was used to determine the distribution (normal or non-normal) of the data. Continuous variables were analyzed using either the unpaired Student’s t-test (data with normal distribution) or the Mann-Whitney test (data with non-normal distribution). Categorical variables were analyzed using Pearson’s chi-squared (χ2) test. All analyses were performed with the aid of the SAS 9.4 software and a p-value < 0.05 was considered indicative of statistical significance.

Interobserver variability was determined by comparing the measures of 20 patients obtained by three different cardiologists. Intra-observer variability was determined by comparing the measures obtained by one cardiologist taken a second time after a one-month interval.

## Results

Among the 100 transplant patients at the institution in the study period, 71 were included, totaling 120 biopsies, with a minimum interval of six days, a maximum of 328 days, and a median of 70 days. However, 35 patients were excluded during the study (1 per limited acoustic view, 1 for inconclusive EMB, 1 due to reactivation of Chagas disease, 1 for ejection fraction less than 53% and 31 for having EMB indicative of rejection), with 36 transplant patients remaining for analysis and compared to 30 healthy individuals in the control group ( Figure 3 ).

**Figure 3:** – Flowchart of Heart Transplant patients from 2017 to 2019 at the Instituto de Cardiologia e Transplante do Distrito Federal. EMB: Endomiyocardial Biopsy; TX: transplanteds.

The chi-squared test revealed no significant differences between the transplant and control groups concerning age or sex. The transplant group had higher mean blood pressure and heart rate. The control group had significantly greater mean weight and body mass index ( Table 1 ).

**Table 1:** – Clinical characteristics of heart transplant groups without rejection and healthy individuals (control)

Clinical Characteristic	Transplanted without rejection (n=36)				Control (n=30)				p-value

	Mean or Median	SD/ QR	QT 1	QT 3	Mean or Median	SD/ QR	QT 1	QT 3	
**Age (years)**	52.50	12.50	44.00	56.50	46.00	7.00	44.00	51.00	0.2450^†^
**Feminine Gender**	17 (40.48%)	-	-	-	25 (59.52%)	-	-	-	0.2826^‡^
**SBP (mmHg)**	122.00	31.00	111.00	142.00	117.00	17.00	111	128	0.1910^†^
**DBP (mmHg)**	83.33	16.94	-	-	69.80	10.15	-	-	0.0002^*^
**HR (bpm)**	83.61	13.97	-	-	75.17	9.55	-	-	0.0051^*^
**Height (cm)**	164.00	13.00	158.00	171.00	165.50	17.00	158	175	0.5706^†^
**Weight (kg)**	60.75	10.01	-	-	72.86	11.87	-	-	<0.0001^*^
**BS (m^2^)**	1.66	0.15	-	-	1.80	0.22	-	-	0.0046^*^
**BMI (cm^2^/kg)**	21.59	3.86	20.26	24.12	26.18	2.92	20.26	24.12	<0.0001^†^

* p-value calculated by Student’s t-test. † p-value calculated by the non-parametric Mann-Whitney test. ‡ p-value calculated by the Chi-square test. QR: Interquartile range; SBP: systolic blood pressure; DBP: diastolic blood pressure; HR: heart rate; BS: body surface; BMI: body mass index; QT: quartile. Note: values expressed as mean and standard deviation, or median and interquartile range/with the intervals of the first and third quartile, or frequency (%)

Among the transplant patients, the donor and recipient were the same sex in 19 cases (52.78%). Most donors were men (63%). Regarding the immunosuppressants, 26 patients were taking tacrolimus (72.22%), 10 were taking cyclosporin (27.78%), 25 were taking mycophenolate (69.44%), 11 were taking azathioprine (30.56%) and 34 were taking prednisone (94.44%). Eight (22.22%) of the transplanted patients had arterial hypertension and nine (25%) had diabetes. Chagas disease was the most prevalent etiology of pre-transplant heart failure (52.77%). The median time between the heart transplant and EMB and echocardiogram was 70 days, with an interquartile range of 63 days ( Table 2 ).

**Table 2:** – Clinical characteristics of heart transplant recipients without rejection

Variables	N
**Interval between heart transplant and biopsy (days)**	70
Quartil 0	7
Quartil 1	11
Quartil 2	70
Quartil 3	169
**Immunosuppressant Drugs**	
Tacrolimus	26 (72.22%)
Cyclosporine	10 (27.78%)
Mycophenolate	25 (69.44%)
Azathioprine	11 (30.56%)
Prednisone	34 (94.44%)
**Clinical features**	
Donors of the same gender as the recipient	19 (52.78%)
DM	9 (25.00%)
Hypertension	8 (22.22%)
**Etiology of Heart Failure**	
Cardiotoxicity	1 (2.77%)
Non-compaction cardiomyopathy	1 (2.77%)
Chagasic cardiomyopathy	19 (52.77%)
Idiopathic cardiomyopathy	6 (16.66%)
Ischemic cardiomyopathy	3 (8.33%)
Postpartum cardiomyopathy	2 (5.55%)
Restrictive Cardiomyopathy	1 (2.77%)
Valvular cardiomyopathy	2 (5.55%)
Tetralogy of Fallot	1 (2.77%)

The transplant patients had larger interventricular septum and posterior wall measurements and, consequently, greater relative wall thickness and ventricular mass. The volume of the left atrium was greater (mean: 38.17 ml/m^2^) and tissue Doppler measures had lower values in comparison to the control group. However, measures involving diastolic function could not be analyzed in more than 50% of the transplant patients due to technical problems (fusion of waves), with the possible loss of reliability of the data ( Table 3 ).

**Table 3:** – Comparison between echocardiographic variables of heart transplant recipients without rejection and healthy volunteers (control)

Echocardiographic Variable*	Transplanted without rejection (n=36)				Control (n=30)				p-value

	Mean or Median	SD/ QR	QT 1	QT 3	Mean or Median	SD/ QR	QT 1	QT 3	
SW (mm)	10.00	10.00	9.00	11.50	8.00	1.00	7.00	8.00	<0.0001^‡^
PW (mm)	9.50	9.50	8.00	11.00	7.50	1.00	7.00	8.00	<0.0001^‡^
LVDD (mm)	43.69	3.09	-	-	44.70	3.20	-	-	0.2011^†^
LVSD (mm)	28.00	28.00	26.50	31.00	28.00	3.00	26.00	29.00	0.5027^‡^
EF Teicholz (%)	65.49	5.23	62.53	67.76	66.91	6.35	64.43	70.78	0.0491^‡^
LVDV Simpson (mL)	74.99	23.00	60.00	83.00	73.00	28.00	63.00	91.00	0.9790^‡^
LVSV Simpson (mL)	26.00	16.00	21.00	37.00	27.00	13.00	20.00	33.00	0.8075^‡^
EF Simpson (%)	64.52	6.88	59.78	66.67	62.90	6.74	60.29	67.03	0.7371^†^
LV Mass Devereux (g)	148.69	44.51	-	-	108.15	25.88	-	-	<0.0001^†^
LV Mass index (g/m^2^)	90.79	29.89	-	-	59.91	11.53	-	-	<0.0001^†^
Relative wall thickness	0.44	0.09	-	-	0.33	0.04	-	-	<0.0001^†^
LA Volume (mL)	61.75	29.50	49.75	79.25	34.50	12.00	30.00	42.00	<0.0001^‡^
LAi volume (mL/ m^2^)	38.17	12.90	31.21	44.11	18.98	7.18	16.29	23.47	<0.0001^‡^
RA Volume (mL)	35.00	19.00	25.00	44.00	27.50	12.00	22.00	34.00	0.0200^‡^
RAi Volume (mL/ m^2^)	19.91	11.62	15.40	27.02	15.61	7.51	11.55	19.07	0.0014^‡^
RV basal (mm)	33.64	5.79	-	-	33.93	3.58	-	-	0.8014^†^
RV medial (mm)	29.11	5.41	-	-	26.33	3.96	-	-	0.0226^†^
Lateral annulus tricuspid SV (cm/s)	7.40	2.06	-	-	13.37	2.08	-	-	<0.0001^†^
TAPSE (mm)	12.51	3.18	-	-	22.97	3.01	-	-	<0.0001^†^
RV FAC (%)	42.85	7.51	-	-	44.37	4.55	-	-	0.3159^†^

* values expressed as mean and standard deviation, or median and interquartile range. † p-value calculated by Student’s t-test. ‡ p-value calculated by the non-parametric Mann-Whitney test. QR: Interquartile Range; SW: left ventricular septum thickness; PW: posterior wall thickness; LVDD: left ventricular diastolic diameter; LVSD: left ventricular systolic diameter; EF: ejection fraction; LVDV: left ventricle diastolic volume; LVSV: left ventricular systolic volume; LA: left atrium; LAi: left atrium indexed by body surface; RA: right atrium; RAi: right atrium indexed by body surface; LV: left ventricle; RV: right ventricle; TAPSE: tricuspid annulus systolic excursion; FAC: fractional shortening; QT: quartile.

In the assessment of RV function, lower RV S and TAPSE values were found in the transplant patients, but FAC was within the range of normality ( Table 3 ). Strain determined by speckle tracking was significantly lower and below reference values^2^in the transplant patients for both LV GLS and RV LS. Myocardial work indices were lower in the transplant patients compared to the control group. Mean MWI, GWE, and GCW were significantly lower in the transplant patients compared to the healthy controls (p < 0.0001) and GWW was greater in the transplant group ( Table 4 ).

**Table 4:** – Comparison of Speckle Tracking Strain and Myocardial Work between transplant patients without rejection and healthy individuals (control group)

Strain / Myocardial Work*	Transplanted without rejection (n=36)				Control (n=30)				p-value

	Mean or Median	SD/ QR	QT 1	QT 3	Mean or Median	SD/ QR	QT 1	QT 3	
LV GLS	11.99	2.740	-	-	20.60	2.15	-	-	<0.0001^†^
RV FWLS	16.67	4.33	18.00	13.67	25.50	7.03	30.33	23.30	<0.0001^‡^
MWI (mmHg %)	1131.69	469.43	-	-	2005.10	339.10	-	-	<0.0001
GEW (%)	85.43	8.70	-	-	96.30	2.07	-	-	<0.0001
GCW (mmHg %)	1395.91	505.62	-	-	2758.50	3529.20	-	-	<0.0001
GWW (mmHg %)	182.11	143.90	-	-	68.55	52.58	-	-	<0.0001

* values expressed as mean and standard deviation, or median and interquartile range. † p-value calculated by Student’s t-test. † p-value calculated by the non-parametric Mann-Whitney test. LV GLS: Left Ventricular Global Longitudinal Strain; RV FWLS: right ventricular free wall strain; MWI: myocardial work index; GEW: Global Efficiency Work; GCW: Global Constructive Work; TGD: Global Wasted Work. QT: quartile.

The intraclass correlation coefficient for LV GLS was 0.98 (95% CI: 0.94-0.99) for interobserver variability and 0.88 (95% CI: 0.70-0.99) for intra-observer variability. The intraclass correlation coefficient for RV LS was 0.97 (95% CI: 0.94-0.95) for interobserver variability and 0.98 (95% CI: 0.95-0.99) for intra-observer variability. Interobserver variability coefficients for myocardial work derived from the GLS were 0.93 (95% CI: 0.84-0.97) for MWI, 0.97 (95% CI: 0.93-0.99) for GWE, 0.94 (95% CI: 0.85-0.97) for GCT and 0.92 (95% CI: 0.81-0.97) for GWW.

## Discussion

The main finding of the present study was the low heart mechanic values (obtained based on strain determined by speckle tracking) and myocardial work in a cohort of transplant patients without rejection in comparison to healthy individuals of similar age and sex. In the transplant patients, low left ventricle global longitudinal strain, free wall strain of the right ventricle, and myocardial work indices were found, along with a larger left atrium size, larger mass, and larger relative wall thickness. Moreover, Chagas disease was the main etiology of heart failure that culminated in heart transplantation.

In agreement with previous studies involving heart transplant patients,^5^the present data reveal ventricular remodeling, with an increase in the LV mass index and relative wall thickness. Badano et al.^15^reported that an increase in left ventricular mass and wall thickness can occur in the first months after heart transplantation, likely caused by cellular inflammatory infiltration and edema of the graft. During follow-up, a secondary increase in LV mass and wall thickness can occur due to multiple factors, such as repeated episodes of rejection (not found in the participants of the present study), chronic tachycardia, ischemic injury and systemic hypertension, which is usually induced by immunosuppressants.^6^

The ejection fraction remained within the range of normality in the transplant group (LVEF: 64.52 ± 6.88%), but the strain indices obtained by speckle tracking were lower (LV GLS: 11.99 ± 2.74) than those in the control group and patterns of normality described in guidelines.^2^

Studies report the strain measures are low in many clinical situations with a preserved ejection fraction.^17 , 18^However, the clinical significance of values below the range of normality for variables in the first year after heart transplantation is not clear. Badano et al.^15^suggest an analysis six months after heart transplantation to determine basal values of normality in such cases when ischemic injury is lower.

The present findings are in agreement with data reported in other studies that found low GLS values after transplantation, even in patients without rejection.^5 , 15^The surgical procedure, initial inflammation, and heart remodeling may contribute to the drop in GLS. Ingvarsson et al.^5^put forth the hypothesis of low strain variables being related to possible heart failure with a preserved ejection fraction in transplant patients, as such patients exhibit changes in ventricular mass and a restrictive pattern of diastolic dysfunction.

Despite the superiority of GLS over LVEF in assessments of the systolic performance of the left ventricle,^14^this technique is limited due to its dependence on ventricular filling, affecting the assessment of myocardial contractile function under specific conditions. To minimize the effect of the afterload of arterial pressure on the strain, myocardial work emerges as a novel echocardiographic tool that enables quantifying LV performance based on the strain/pressure curve. The present investigation is the first study in the literature to describe myocardial work in heart transplant patients.

Reductions in myocardial work indices and an increase in wasted work were found in the transplant group. These data confirm that the transplanted heart – even without rejection – has a poorer performance, with greater wasted work. The hypothesis for this regards the previously described changes in heart dynamics after surgery or an initial state of inflammation.^15^

Indices for the assessment of right ventricular function, such as RV S, TAPSE, and free wall strain, were lower in the transplanted group compared to the control group (p <0.0001), whereas the shortening fraction and ventricular size remained normal. Previous studies described similar results.^5^One should bear in mind that TAPSE and RV S are ineffective at differentiating active contraction from passive contraction caused by the left ventricle and, therefore, do not reflect true RV function. Raina et al.^19^suggest that chest surgery alters the contractability of the right ventricle, diminishing longitudinal contraction and increasing radial contraction. This finding was confirmed in an experimental study using the brains of donors, in which the increase in intracranial pressure and catecholamine levels were related to the reduction in RV function after heart transplantation.^20^Thus, the quality of donor maintenance, explant technique, time of ischemia, hypothermia, and implant in the recipient have impacts on RV function after transplantation.^5^

In the present study, a significant increase in left atrium size was found although the bicaval surgical technique was used in all transplant patients rather than the biatrial technique, which is older and has the characteristic of leading to larger atria. The presence of larger atria despite the use of the bicaval technique may indicate a disproportion in the size of the graft from the donor concerning the recipient, resulting in a volume indexed by the larger body surface or even progressive diastolic stiffening with the increase in filling pressures, causing diastolic dysfunction and an increase in atrium size.^1^

Unlike descriptions in previous studies, the main reason for transplantation in the present sample was Chagas disease (more than 50%). However, no reactivation of the disease was found in the patients studied. Moreover, the small number of patients impedes the assessment of differences in conventional echocardiographic variables and heart mechanics related to the presence of this disease and other causes of heart failure, as also demonstrated by Otto et al.^7^

The present study has some limitations that should be considered. The study was developed at a single center. No information was collected on the characteristics of the donors, as many organs came from regions distant from the research center. The sample size was small and only one type of software was used for the assessment. Strain determined by speckle tracking and myocardial work are evolving tools, and the values obtained in this study cannot be applied to other strain analysis software programs. A multicentric study involving different cardiogram equipment and a larger number of patients is needed.

As a strong point, this is the first study to assess myocardial work in transplant patients without rejection and to find different heart mechanics from that seen in a comparable group of healthy individuals.

## Conclusion

In the first year after heart transplantation, patients without rejection proved by EMB exhibited lower global longitudinal strain of the left ventricle, lower free wall strain of the right ventricle, and lower myocardial work, but a similar ejection fraction to that found in the comparable group of healthy individuals. Moreover, an increase in the size of the left atrium and greater remodeling of the left ventricle were found in the transplant patients. These findings indicate that the pattern of echocardiographic variables differs between transplant patients without rejection and healthy individuals, which can be clinically relevant during the follow-up of these patients.

For the determination of reference values of specific echocardiographic measures for heart transplant patients, multicentric studies with a larger number of patients are needed.
